# Data regarding covariates significantly associated with sarcopenia and varying albumin statuses in patients with renal cell carcinoma

**DOI:** 10.1016/j.dib.2022.108724

**Published:** 2022-11-04

**Authors:** Benjamin N. Schmeusser, Dattatraya H. Patil, Eric Midenberg, Michelle I. Higgins, Joel Zaldumbide, Dylan J. Martini, Sean Steele, Milton Williams, Reza Nabavizadeh, Sarah P. Psutka, Kenneth Ogan, Mehmet Asim Bilen, Vraj A. Master

**Affiliations:** a Department of Urology, Emory University School of Medicine, Atlanta, GA, USA; b Brady Urological Institute, Johns Hopkins Hospital, Baltimore, MD, USA; c Department of Medicine, Massachusetts General Hospital, Boston, MA, USA; d Department of Urology, University of Alabama, Birmingham, AL, USA; e Department of Urology, University of Washington, Seattle, WA, USA; f Fred Hutchinson Cancer Center, University of Washington, Seattle, WA, USA; g Department of Hematology and Medical Oncology, Emory University School of Medicine, Atlanta, GA, USA; h Winship Cancer Institute of Emory University, Atlanta, GA, USA

**Keywords:** Body composition, muscle mass, sarcopenia, cancer outcomes, malnutrition

## Abstract

Poor functional, nutritional, and muscle status is a significant negative predictor for surgical and survival outcomes in patients with cancer, including renal cell carcinoma. This dataset displays results from preoperative muscle composition analysis and albumin levels in a large cohort (*n* = 473) of patients undergoing surgery for renal cell carcinoma. Data was obtained from retrospective review of prospectively maintained databases and retrospective image analysis. The optimal cut-point for skeletal muscle index (sarcopenia) was determined by a receiver operatic characteristic analysis to optimally stratify cohort, adjusting for BMI and sex. A threshold value of 3.5 g/dL was used to categorize normal versus low serum albumin. Patients were stratified into low risk (non-sarcopenic and normal albumin), medium risk (non-sarcopenic and low albumin, or sarcopenic and normal albumin), and high risk (sarcopenic and low albumin) groups. This data could potentially be used in future studies to determine other relationships between nutrition and musculature in renal cell carcinoma patients.

**Specifications Table**

**Table:** 

Subject	Health and Medical Sciences
Specific subject area	Urology
Type of data	Table
How the data were acquired	Data was obtained via retrospective chart review of prospectively maintained databases.
Data format	Filtered (Protected health information removed)
Description of data collection	This dataset was compiled from prospectively maintained databases for patients presenting to a tertiary referral center for renal cell carcinoma. Out of the patients included in the database, retrospective review and skeletal muscle calculation was completed on patients that underwent nephrectomy, had appropriate imaging obtained ≤100 days preoperatively, and had preoperative albumin levels.
Data source location	Data collected from patients presenting to Emory University medical center, Atlanta, Georgia, USA. Patients presenting are from Georgia and neighboring states of Alabama, Tennessee, and South/North Carolina's, and Florida.
Data accessibility	All of the data is provided in this article and within the data repository listed below. Repository name: Mendeley Data Data identification number: 10.17632/pytnxsgt3k.1 Direct URL to data: https://data.mendeley.com/datasets/pytnxsgt3k/1
Related research article	E. Midenberg, M.I. Higgins, B. Schmeusser, D.H. Patil, J. Zaldumbide, D.J. Martini, S. Steele, M. Williams, R. Nebavizadeh, S. P. Psutka, K. Ogan, V.A. Master. Prognostic Value of Sarcopenia and Albumin in the Surgical Management of Localized Renal Cell Carcinoma, *Urologic Oncology: Seminars and Original Investigations.* In press. https://doi.org/10.1016/j.urolonc.2022.09.020.

## Value of the Data

• As interest in how muscle mass and nutritional status impact health, surgical, and survival outcomes continues to grow, this information provides insight into associated factors.
• Understanding the effects of nutrition and muscle composition on patient care and outcomes is useful for any clinical professional.
• Use of this data may allow clinicians to identify patients that may potentially benefit from pretreatment intervention, such as exercise and nutrition.
• Patients will benefit from this information as future care may be more personalized based on nutritional, functional, and muscle status.
• Researchers and clinicians may use this data to relate to their patient populations, as well as represent an example of possible future research.

## Objective

The dataset was generated to further increase our understanding of how nutrition status and muscle composition impact outcomes in patients with renal cell carcinoma (RCC). Specifically, this data aimed to not only validate malnutrition and sarcopenia as poor prognosticators in RCC, but to additionally examine how they are associated with each other and their combined impact. This dataset adds value to the associated published article in that it details the patient characteristics associated with malnutrition and sarcopenia in our patient cohort. Other researchers and clinicians may use this data to further their understanding of the potential impact of nutritional and muscle status, as well as to help them identify patients that may be at risk of being malnourished or sarcopenic.

## 1 Data Description

This dataset describes 473 patients that underwent partial or radical nephrectomy for non-metastatic RCC at a tertiary academic referral center. Included patients must have had skeletal muscle index (SMI) measured on computed tomography (CT) and magnetic resonance imaging (MRI) obtained ≤100 days preoperatively, as well as preoperative albumin values.

The referenced data has been de-identified and anonymized to protect patient confidentiality. The dataset contains information on the following variables for all subjects included in the analysis: sarcopenia as a binary variable, sarcopenia and albumin combination variable, age greater than or equal to 60 years, subject gender, Eastern Cooperative Oncology Group (ECOG) as a binary variable at the time of presentation, obesity (body mass index [BMI] greater than or equal to 30kg/m^2^), BMI as a categorical variable, Fuhrman nuclear grade (aka RCC grade) based on histological exam, Fuhrman nuclear grade as a binary variable, T-Stage of tumor, SSIGN as a categorical variable, follow-up end-point of death at the time of last follow-up of patient, and first known tumor recurrence after surgery.

Tables 1 and 2 display the results for the cohort stratified by the presence of sarcopenia alone and by sarcopenia and albumin risk groups. Analysis of variances (ANOVA) determined significant associations between covariates. Table 1 summarizes the cohort by presence of sarcopenia alone, with Age ≥60, obesity (BMI≥30 kg/m^2^), nuclear grade, and Fuhrman Grade 3-4 disease being significantly associated with the presence of sarcopenia. Table 2 summarizes the cohort by sarcopenia and albumin risk groups. Age ≥60, male sex, ECOG 0, obesity, nuclear grade, pathologic T1-T2 disease, Fuhrman Grade 3-4, and SSIGN score were significantly associated with combined sarcopenia and hypo-albuminemia.

**Table 1:** Cohort summary by Sarcopenia status.

	Sarcopenia		
Covariate	No (n [%],*n* = 272)	Yes (n[%],*n* = 201)	*p*-value
**Age at Surgery ≥ 60**	127 (46.7)	133 (66.2)	<0.001
**Obesity (BMI≥30 kg/m^2^)**	138 (50.7)	70 (34.8)	<0.001
BMI (kg/m^2^)*	30.0 (12.5-75.0)	26.5 (15.7-44.6)	<0.001
**Nuclear Grade**			
1	6 (2.2)	3 (1.5)	<0.040
2	101 (37.4)	57 (28.8)	
3	120 (44.4)	87 (43.9)	
4	43 (15.9)	51 (25.8)	
**Fuhrman Grade**			
1-2	107 (39.6)	60 (30.3)	0.037
3-4	163 (60.4)	138 (69.7)	

**Abbreviations:** BMI, body mass index; RCC, Renal Cell Carcinoma;

⁎ = Median (min-max)

**Table 2:** Cohort summary of risk groups by preoperative Sarcopenia and albumin status.

	Sarcopenia and Albumin Status (No [%])				
Covariate	Low Risk: No Sarcopenia and Normal Albumin (*n* = 214)	Medium Risk: Sarcopenia Only (*n* = 143)	Medium Risk: Low Albumin Only (*n* = 58)	High Risk: Sarcopenia and Low Albumin (*n* = 58)	*p*-value
**Age at Surgery ≥ 60**	99 (46.3)	100 (69.9)	28 (48.3)	33 (56.9)	<0.001
**Sex**					
Male	151 (70.6)	90 (62.9)	29 (50)	42 (72.4)	0.017
**ECOG Status**					
ECOG=0	192 (90.1)	125 (89.3)	43 (74.1)	40 (69)	<0.001
**Obesity (BMI≥30 kg/m^2^)**	114 (53.3)	56 (39.2)	24 (41.4)	14 (24.1)	<0.001
BMI (kg/m^2^)*	30.6 (12.5-75)	27.3 (17.2-44.6)	28.3 (20.7-58.4)	25.5 (15.7-40)	<0.001
**Nuclear Grade**					
1	5 (2.4)	1 (0.7)	1 (1.7)	2 (3.5)	<0.001
2	80 (37.7)	48 (34)	21 (36.2)	9 (15.8)	
3	101 (47.6)	64 (45.4)	19 (32.8)	23 (40.4)	
4	26 (12.3)	28 (19.9)	17 (29.3)	23 (40.4)	
**Pathologic T-stage**					
T1-T2	119 (55.6)	77 (53.8)	28 (48.3)	20 (34.5)	0.033
T3-T4	95 (44.4)	66 (46.2)	30 (51.7)	38 (65.5)	
**Fuhrman Grade**					
1-2	85 (40.1)	49 (34.8)	22 (37.9)	11 (19.3)	0.034
3-4	127 (59.9)	92 (65.2)	36 (62.1)	46 (80.7)	
**SSIGN Score**					
0-2	99 (46.3)	57 (39.9)	22 (37.9)	15 (25.9)	<0.001
3-5	65 (30.4)	47 (32.9)	12 (20.7)	12 (20.7)	
≥6	50 (23.4)	36 (27.3)	24 (41.4)	31 (53.4)	

**Abbreviations:** BMI, body mass index; ECOG, Eastern Cooperative Oncology Group; SSIGN, Stage, Size, Grade and Necrosis; RCC, Renal Cell Carcinoma;

⁎ Median (Min-Max)

## 2 Experimental Design, Materials and Methods

### 2.1 Study Population and Data Collection

This study consisted of a retrospective review of a prospectively maintained database. Patients that underwent radical or partial nephrectomy from June 2004 to October 2020 were identified. Primary inclusion/exclusion criteria of interest were nonmetastatic disease at the time of surgery, histological identification of RCC, preoperative CT or MRI of the abdomen and/or pelvis within 100 days prior to surgery, and albumin levels within 100 same time-frame. Age, sex, race, BMI, ECOG score, TNM staging, nuclear grade, Fuhrman Grade, and SSIGN scores were incorporated into the analysis.

### 2.2 Skeletal Muscle Index Calculation

Preoperative axial CT and MRI images were segmented at the mid-L3 vertebral level as previously described.[1,2] Imaging segmentation was completed by 4 trained observers following training requiring <1% inter-observer and intra-observer variability. Each observer was blinded to patient history and outcomes. Image processing was completed using Slice-O-Matic software (version 5.0; Tomovision) using predesignated thresholds for skeletal muscle (-29 to +150 Hounsfield units for CT and region growing tool for MRI).[1], [2], [3], [4] Skeletal muscle area (SMA) in cm^2^ was determined by measuring the combined surface area of the psoas, quadratus lumborum, erector spinae, transversus abdominis, internal oblique, external oblique, and rectus abdominis. Skeletal muscle index (SMI; cm^2^/m^2^) was calculated by normalizing the SMA by height in meters squared.

### 2.3 Statistical Analysis

To determine the SMI threshold that delineated the presence or absence of sarcopenia, a receiver operatic characteristic (ROC) analysis and grid search best fit method was performed to optimally stratify our patient population while adjusting for BMI and sex, as done in previous studies.[4], [5], [6] For patients with a BMI<30 kg/m^2^, sarcopenia was defined as a SMI<47 cm^2^/m^2^ for males and a SMI<38 cm^2^/m^2^ for females. For patients with a BMI≥30 kg/m^2^, sarcopenia was defined as a SMI<54 cm^2^/m^2^ for males and a SMI<47 cm^2^/m^2^ for females. A threshold value of 3.5 g/dL was used to categorize normal versus low serum albumin.

To analyze the effects of sarcopenia and serum albumin together, patients were stratified into low risk (non-sarcopenic and normal albumin), medium risk (non-sarcopenic and low albumin, or sarcopenic and normal albumin), and high risk (sarcopenic and low albumin) groups. Patients in the medium risk category were analyzed separately depending on the presence of sarcopenia (medium risk-sarcopenia) or hypoalbuminemia (medium risk-hypoalbuminemia). Patient factors such as age, BMI, ECOG score, were collected at the time of presentation for the surgery. Tumor factors such as TNM-stage, RCC grade, histological tumor type, and necrosis were based on tissue examination by an expert pathologist.

All follow-up outcomes were collected based on an IRB approved protocol, by either reviewing patient charts for any follow-up visits and treatments received after surgery, or in case of no records searching for patient details in a National Death index / Social security death index maintained by CDC and state department of public health. RCC recurrence was defined based on earliest known evidence of tumor recurrence after surgery. The confirmation evidence accepted was either confirmed imaging, histological, or receipt of adjuvant and/or salvage surgery based on approved treatment protocols. Time to recurrence or death from any cause was calculated from surgery to outcome event or last known follow-up of the subject in medical records.

## Ethics Statements

In this dataset, relevant informed consent was obtained from all subjects. The collection of this data was carried out in accordance with the Declaration of Helsinki and received approval by the Emory Institutional Review Board (IRB00055316).

## CRediT authorship contribution statement

**Benjamin N. Schmeusser:** Conceptualization, Methodology, Investigation, Writing – original draft, Writing – review & editing. **Dattatraya H. Patil:** Methodology, Validation, Data curation, Formal analysis, Writing – original draft, Writing – review & editing, Visualization. **Eric Midenberg:** Conceptualization, Methodology, Investigation, Writing – original draft, Writing – review & editing. **Michelle I. Higgins:** Conceptualization, Methodology, Investigation, Writing – original draft, Writing – review & editing. **Joel Zaldumbide:** Investigation, Writing – original draft, Writing – review & editing. **Dylan J. Martini:** Investigation, Writing – original draft, Writing – review & editing. **Sean Steele:** Investigation, Writing – original draft, Writing – review & editing. **Milton Williams:** Investigation, Writing – original draft, Writing – review & editing. **Reza Nabavizadeh:** Investigation, Writing – review & editing, Visualization. **Sarah P. Psutka:** Investigation, Conceptualization, Writing – review & editing, Supervision. **Kenneth Ogan:** Investigation, Conceptualization, Writing – review & editing, Supervision. **Mehmet Asim Bilen:** Investigation, Conceptualization, Writing – review & editing, Supervision. **Vraj A. Master:** Investigation, Conceptualization, Writing – review & editing, Supervision, Project administration, Funding acquisition.

## Declaration of Competing Interest

The authors have no conflicts of interest to disclose except for the following. Mehmet Asim Bilen has acted as a paid consultant and/or as a member of the advisory boards of Calithera Biosciences, Exelixis, Bayer, Bristol-Myers Squibb, Eisai, Pfizer, AstraZeneca, Janssen, Genomic Health, Nektar, and Sanofi and has received grants for his institution from Xencor, Bayer, Bristol-Myers Squibb, Genetech/Roche, Seattle Genetics, Incyte, Nektar, AstraZeneca, Tricon Pharmaceuticals, Genome & Company, AAA, Peleton Therapeutics, and Pfizer for work performed outside the current project.

## Data Availability

Data Regarding Covariates Significantly Associated with Sarcopenia and Varying Albumin Statuses in Patients with Renal Cell Carcinoma (Original data) (Mendeley Data).

## Appendix Supplementary materials

Image, application 1

## References

[1] Steele S., Lin F., Le T.-L., Medline A., Higgins M., Sandberg A., Evans S., Hong G., Williams M.A., Bilen M.A., Psutka S., Ogan K., Master V.A. (2021). Segmentation and Linear Measurement for Body Composition Analysis using Slice-O-Matic and Horos. J. Vis. Exp..

[2] Khan A.I., Psutka S.P., Patil D.H., Hong G., Williams M.A., Bilen M.A., Sekhar A., Kissick H.T., Narayan V.M., Joshi S.S., Ogan K., Master V.A. (2022). Sarcopenia and systemic inflammation are associated with decreased survival after cytoreductive nephrectomy for metastatic renal cell carcinoma. Cancer.

[3] Psutka S.P., Boorjian S.A., Moynagh M.R., Schmit G.D., Costello B.A., Thompson R.H., Stewart-Merrill S.B., Lohse C.M., Cheville J.C., Leibovich B.C., Tollefson M.K. (2016). Decreased skeletal muscle mass is associated with an increased risk of mortality after radical nephrectomy for localized renal cell cancer. J. Urol..

[4] Higgins M.I., Martini D.J., Patil D.H., Nabavizadeh R., Steele S., Williams M., Joshi S.S., Narayan V.M., Sekhar A., Psutka S.P., Ogan K., Bilen M.A., Master V.A. (2021). Sarcopenia and modified Glasgow Prognostic Score predict postsurgical outcomes in localized renal cell carcinoma. Cancer.

[5] Caan B.J., Meyerhardt J.A., Kroenke C.H., Alexeeff S., Xiao J., Weltzien E., Feliciano E.C., Castillo A.L., Quesenberry C.P., Kwan M.L., Prado C.M. (2017). Explaining the obesity paradox: The Association between Body Composition and Colorectal Cancer Survival (C-SCANS Study). Cancer Epidemiol. Biomarkers Prev..

[6] Feliciano E.M.C., Kroenke C.H., Meyerhardt J.A., Prado C.M., Bradshaw P.T., Kwan M.L., Xiao J., Alexeeff S., Corley D., Weltzien E., Castillo A.L., Caan B.J. (2017). association of systemic inflammation and Sarcopenia with survival in nonmetastatic colorectal cancer: results From the C SCANS Study. JAMA Oncol.

